# Occipital Bone Erosion Caused by C1 Lateral Mass Screw-Rod Construct Used for C1/2 Arthrodesis: A Case Report

**DOI:** 10.7759/cureus.95257

**Published:** 2025-10-23

**Authors:** Koichiro Okuyama, Genki Tojo, Tadato Kido, Chiaki Sato

**Affiliations:** 1 Orthopedic Surgery, Akita Rosai Hospital, Odate, JPN

**Keywords:** atlantoaxial arthrodesis, c1 lateral mass screw, nuchal crepitus, occipital bone erosion, screw-rod construct

## Abstract

C1 lateral mass screw (C1LMS) is used as a standard implant for fixation of the upper cervical spine. The most significant benefit of the fixation using C1LMS is that it can obtain an excellent mechanical stability immediately after surgery without a massive structural bone graft and strict orthosis. Meanwhile, several studies have already referred to complications, which have not been seen in conventional devices. As for one of the complications, occipital bone erosion (OcBE) induced by repetitive impingement between the occipital bone and the protruded metal implant on the C1 posterior has been rarely reported. OcBE develops infrequently and leads to serious complications, including cerebrospinal fluid leakage and intracranial hemorrhaging. A 78-year-old male case with OcBE, which developed after atlantoaxial arthrodesis using C1 lateral mass and C2 translaminar screws, was presented. In the current case, increased O-C2 angle compensating the subaxial kyphosis, protrusion of C1LMS-rod construct, and cephalad located entry point of C1LMS are related to the occurrence of OcBE.

## Introduction

Nowadays, the C1 lateral mass screw (C1LMS) is used as a standard implant for fixation of the upper cervical spine in which instability, deformity, pain, and neurological impairment intricately exist. The most significant benefit of the fixation using C1LMS is that it can obtain an excellent mechanical stability immediately after surgery without massive structural bone graft and strict orthosis compared to conventional methods using sublaminar wiring and/or laminar hook. The patient underwent surgery of the upper cervical spine, with C1LMS being allowed to have earlier bed leave. This facilitates rehabilitation programs and discharge from the hospital; thus, a better clinical outcome and lower medical costs could be promised. Meanwhile, several studies have already referred to complications, which have not been seen in conventional devices, following the usage of C1LMS. As one of the late complications, occipital bone erosion (OcBE) induced by the protruded screw-rod construct of C1LMS (C1LMS-R-Con) above the atlas has been reported. At maximum cervical extension, the space between the C1 posterior arch and the occipital bone becomes narrowed. OcBE develops by repetitive impingement between the occipital bone and the protruded metal implant on the C1 posterior arch [1]. C1LMS-R-Con has a higher profile than the conventional ones; therefore, it could be presumed that C1LMS-R-Con more easily impinges against the occipital bone. Plant et al. reported the first case of OcBE in 2010 [2], but the prevalence of OcBE is still unknown as far as we are concerned. The common initial symptom of OcBE is occipital pain and/or crepitus. OcBE is associated with tinnitus and dizziness caused by the compressive force of C1LMS-R-Con onto the cerebellum and is infrequently related to more serious complications, including cerebrospinal fluid leakage and intracranial hemorrhaging [3]. Thus, the early detection and treatment of OcBE caused by C1LMS-R-Con are very significant. We present a case of OcBE with an intraoperative finding, which has been rarely demonstrated in previous reports, and a discussion of the pathogenesis of the OcBE. In the current case, implant removal was necessary to avoid further serious complications.

## Case presentation

**Figure 1:**
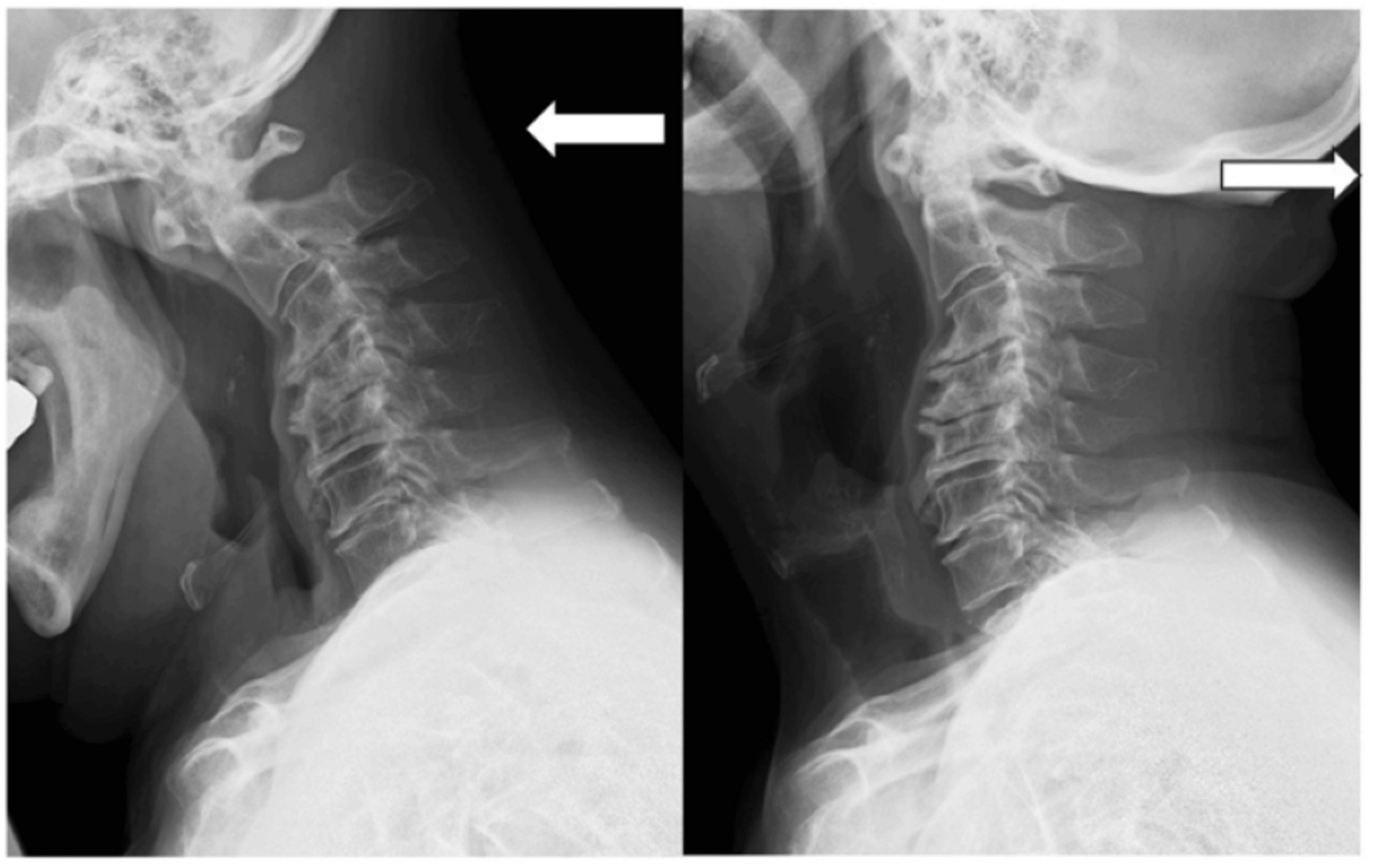
Preoperative dynamic lateral radiograms Demonstrating a 20° degenerative kyphotic change in C3/4/5, an atlantodental interval of 9 mm in flexion, and no space between the occiput bone and the C1 posterior arch in extension (← in white, flexion; → in white, extension).

A 78-year-old male sook medical attention for an unstable sensation while walking for several months. His body height and weight were 167 cm/64 kg, and he had no association with any serious medical co-morbidities. On the initial physical examination, motion pain and tenderness were not found in the cervical spine. Neither neurological deficit nor hyperreflexia was observed, but his gait status was ataxic, and Romberg’s sign was positive. The lateral radiograms demonstrated a degenerative change of a 20° kyphosis in C3/4/5, an atlantodental interval of 9 mm in flexion, and no space between the occiput bone and the C1 posterior arch in extension (Figure 1).

**Figure 2:**
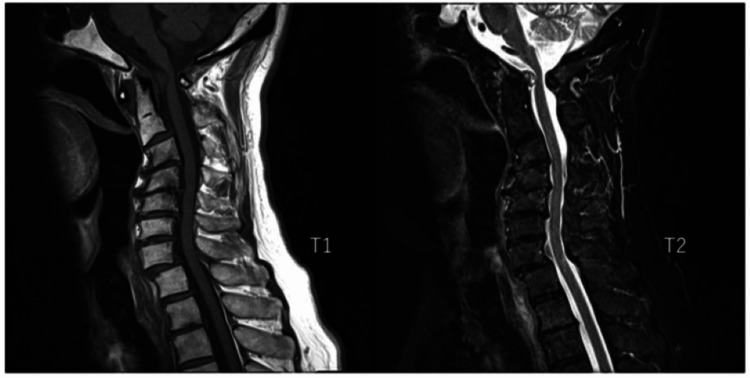
Preoperative MRI A retro-odontoid mass lesion with an iso- and a high-intensity in T1- and T2-weighted images, respectively.

MRI showed a retro-odontoid mass lesion with an iso- and a high-intensity in T1- and T2-weighted images, respectively. The spinal cord was remarkably compressed by the mass lesion (Figure 2).

**Figure 3:**
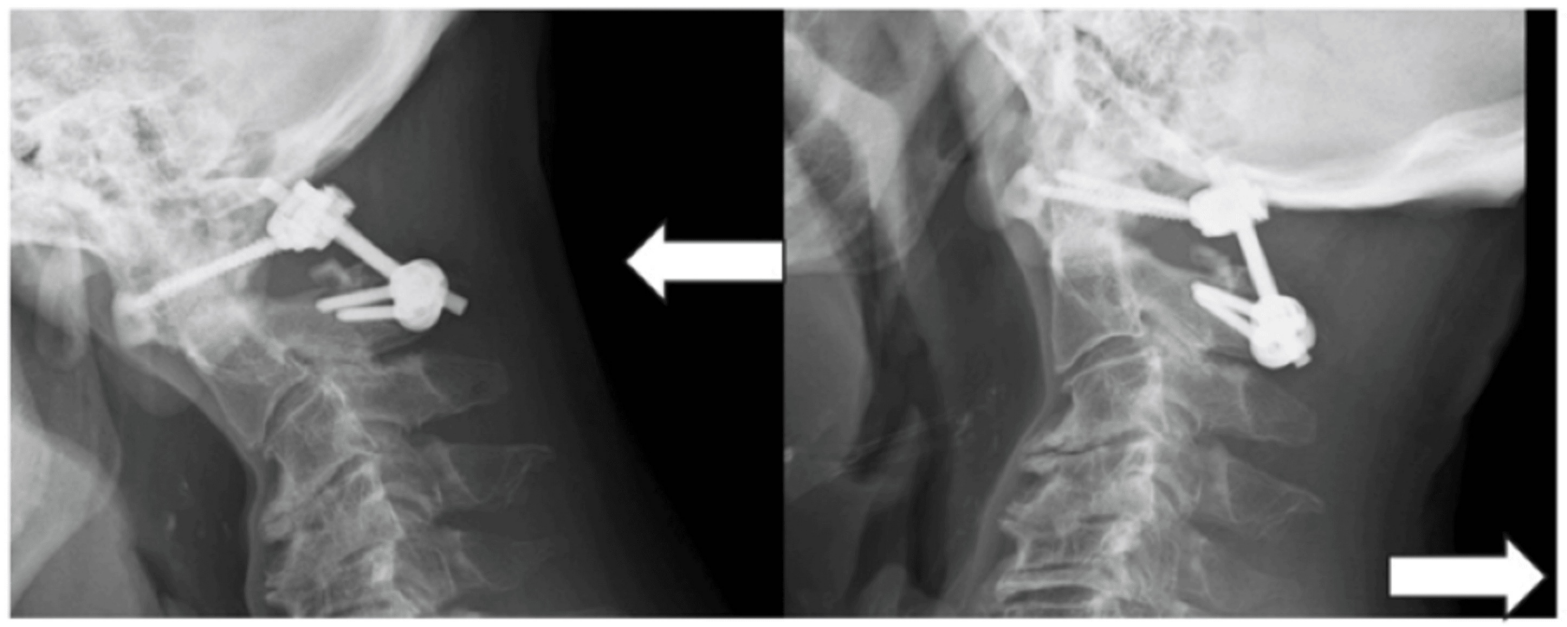
Postoperative dynamic lateral radiograms one year after surgery No dynamic motion of C1/2, but migration of the C1LMS-R-Con into the occipital bone (C1LMS-R-Con, C1 lateral mass screw-rod construct; ← in white, flexion; → in white, extension).

We diagnosed that his gait disturbance was attributed to myelopathy caused by a pseudotumor and atlantoaxial instability. Surgical intervention was selected based on informed consent from the patient. During the surgery, his head was set in a ring under general anesthesia with endotracheal intubation, and then gentle manipulation of C1/2 was done under fluoroscopic control. Reduction of the atlantodental interval was obtained. The posterior elements of the vertebrae from C1 to C4 were bilaterally exposed by subperiosteal dissection through a standard posterior midline approach. Laminectomy of C1 was done with a width of approximately 30 mm. C1LMS, C2 translaminar screw of polyaxial-head type, and rod were bilaterally used. The entry points for C1LMS were placed on the posterior arch of C1 according to Tan et al.’s method [4]. Artificial bone (1 mL) made from hydroxyapatite mixed with collagen and morselized local bone was grafted to the bilateral C1-C2 spaces after decortication. Finally, the wound was closed in layers with an inserted subcutaneous drain. During the operation, no neurological events were recorded on motor-evoked spinal monitoring. The postoperative radiograms showed that the C1LMS-R-Con was almost touching the outer cortex of the occipital bone. The postoperative course was also uneventful. A soft cervical collar was applied for six weeks, but he began to state that he felt a nuchal crepitus without pain while moving his neck several months after the surgery. At that moment, the protruded end of the C1LMS-R-Con was slightly sticking into the occipital bone on the lateral radiograms. After careful continued observation, one year following the surgery, his unstable sensation was alleviated. The MRI showed a shrinkage of the mass and the recovery of spinal cord deformity. However, the nuchal crepitus was unchanged. The dynamic lateral radiograms demonstrated no definitive instability of C1/2, but the C1LMS-R-Con was migrating into the occipital bone (Figure 3).

**Figure 4:**
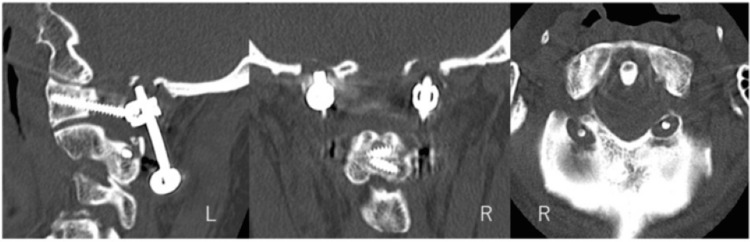
Multiplanar reconstruction computed tomography (MPR-CT) Unveiling bilateral remarkable bony erosion around the C1LMS-R-Con (C1LMS-R-Con, C1 lateral mass screw-rod construct; R, right side; L, left side).

The multiplanar reconstruction computed tomography (MPR-CT) unveiled bilateral remarkable OcBE around the C1LMS-R-Con (Figure 4).

**Figure 5:**
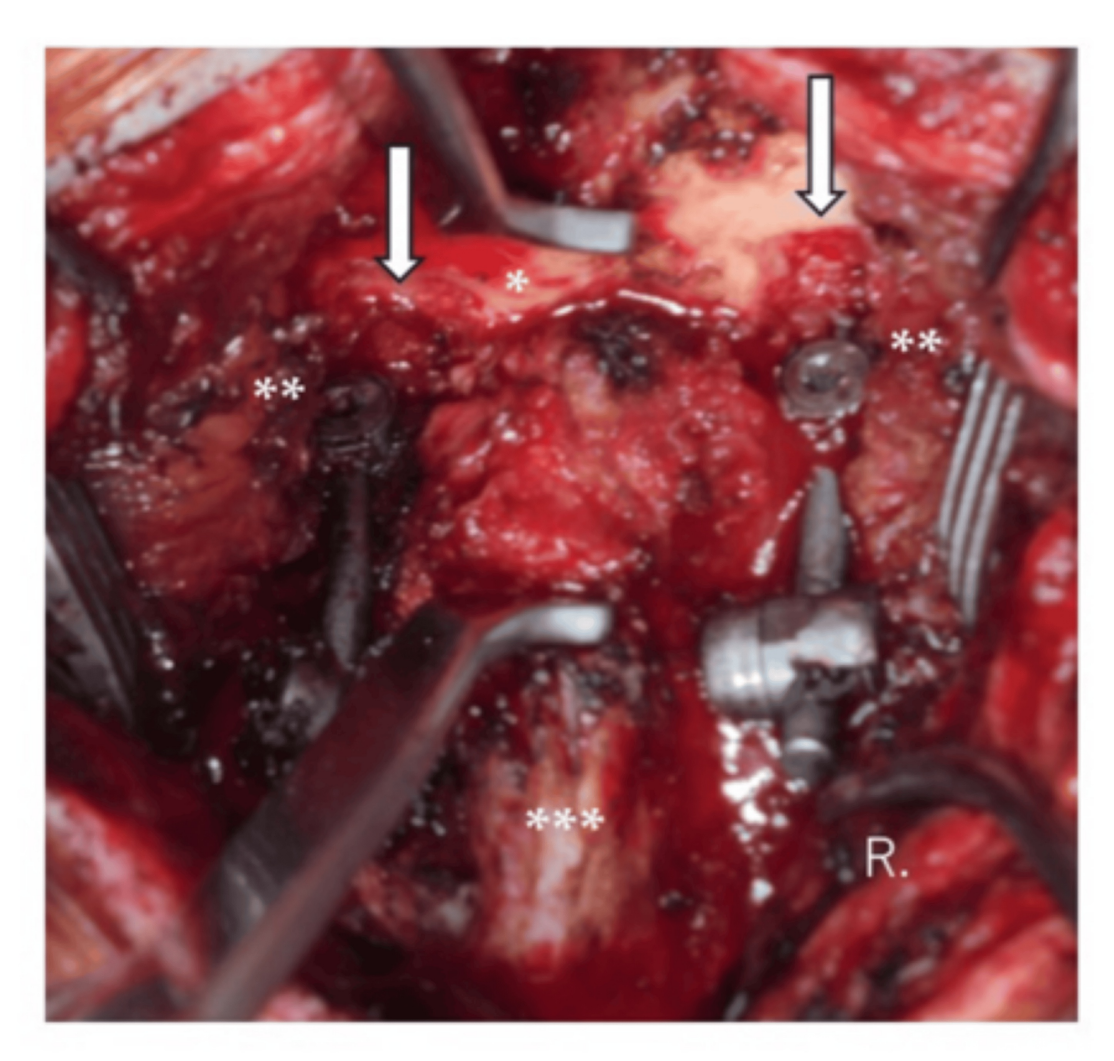
Intraoperative photogram The outer cortex of the occipital bone was bilaterally eroded by the C1LMS-R-Con (C1LMS-R-Con, C1 lateral mass screw-rod construct; *, the occipital bone; **, the C1LMS-R-Con; ***, the lamina of C2; ↓ in white, occipital bone erosion).

We informed the patient that the nuchal crepitus was originating from impingement between the occipital bone and the C1LMS-R-Con and decided to remove the implants to prevent further migration into the skull, which could possibly develop serious complications. Implant removal was done 1.3 years after the first surgery. It was found that not only the protruded rod, but also the ends of C1LMS-R-Con bilaterally had gotten stuck in the occipital bone. They could not be easily retrieved without distracting the space between the C2 lamina and the occipital bone. The inner cortex of the occipital bone was bilaterally breached as well, and the dura mater could be seen at the bottom of the OcBE. Dural damage and leakage of the cerebrospinal fluid were not observed (Figure 5). 

**Figure 6:**
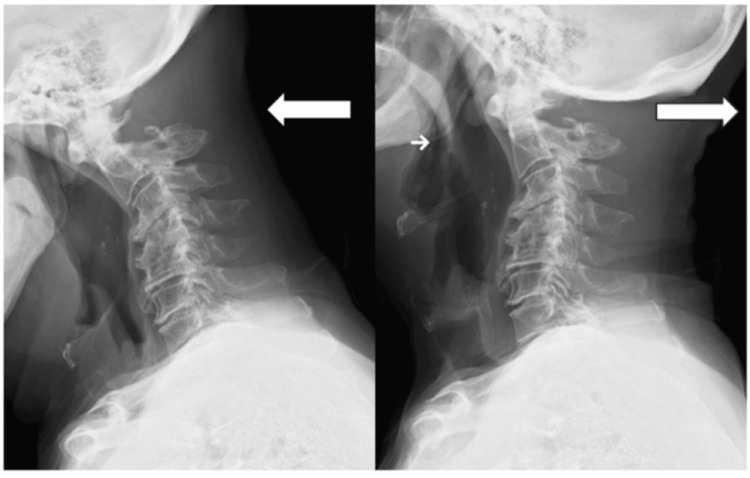
Dynamic lateral radiograms after implant removal No definitive instability of C1/2 (← in white, flexion; → in white, extension).

His nuchal crepitus disappeared immediately after the second surgery without neurological deterioration. Radiological instability of C1/2 was not observed after removal of the implants (Figure 6).

## Discussion

A case with OcBE after atlantoaxial arthrodesis using C1LMS and C2 translaminar screw was reported. Three errors of the C1/2 fixation could be retrospectively pointed out regarding the occurrence of the OcBE in the current case. The overlooking of the increased O-C2 angle compensating subaxial kyphosis, the protruded ends of C1LMS-R-Con, which could have been technically avoided, and the cephalad located entry point of C1LMS should be addressed. Increased O-C2 angle has been mentioned as one of the risk factors of OcBE [5]. The normal value of the O-C2 angle is averaged at 15.8±7.15° [6]. In general, degenerative kyphosis in the subaxial cervical spine increases the O-C2 angle by compensation to maintain horizontal viewing in patients. The increased O-C2 angle means narrowing of the distance between the occipital bone and the posterior arch of C1 and inevitably results in more frequent impingement by C1LMS-R-Con on the surface of the occipital bone. The current case has also demonstrated an increased O-C2 angle of 31° associated with a 20° kyphosis in C3/4/5 before the first surgery. The profile of C1LMS-R-Con is another risk factor of OcBE occurrence. Arizumi et al. have reported that the mean length of the protruded rod above the atlas was 3.5 mm (range: 3.3-4.0) in their cases who had developed OcBE [1]. Another case report recommends that the length of the cephalad protruded rod end should be less than 2 mm to prevent OcBE occurrence [7]. In the current case, the length of the protruded rod of the C1LMS-R-Con was approximately 3.0 mm on MRP-CT. The length of the unnecessary protruded rod should have been as short as possible, or the rod exchanged for an appropriate one. The entry point of C1LMS is also very significant. The cephalad located entry point of C1LMS is also a risk factor. In Goel et al.’s method [8], the entry point of C1LMS was directly on the lateral mass of C1. Contrarily, the entry point was placed on the posterior arch of C1 in Tan et al.’s method. The distance between C1LMS-R-Con and the occipital bone is theoretically shorter in Tan et al.’s [4] method than in Goel’s method. To avoid bleeding from the venous plexus and C2 nerve root damage, C1LMS was inserted according to Tan et al.’s method in the current case. If the C1LMS had been inserted with Goel et al.’s method, the OcBE might have been avoided. In addition, the C2 translaminar screw was used to avoid vertebral artery damage as the bilateral high-riding vertebral artery was suspected in the current case. It is usually inserted more dorsal site of the C2 lamina compared to the C2 pedicle screwing entry point. There is a possibility that the usage of the C2 translamina screw also affected C1LMS-R-Con protrusion. The common initial symptom of OcBE is occipital pain and/or crepitus. OcBE is infrequently associated with tinnitus and dizziness caused by the compressive force on the cerebellum. It is rarely related to more serious complications, including cerebrospinal fluid leakage and intracranial hemorrhaging [3]. Meanwhile, spontaneous bony shell formation around OcBE has also been reported [9]. Latency to the second surgery for OcBE ranges from one month to eight years among sources [5]. Thus, the indication and the timing of removal of the protruded implant or the salvage surgery are very controversial. The decision-making for the second surgery should be done based on the status of neurological findings, bony fusion of C1/2, and the size and depth of C1LMS-R-Con into the skull. In the current case, it intruded more than 3 mm into the skull on MPR-CT and touched the cerebellum at one year after the first surgery. As cerebrospinal fluid leakage, neurological impairment, infection, and cerebral hemorrhage are highly anticipated, removal of the implants was selected based on the informed consent of the patient.

## Conclusions

Repetitive impingement between the occipital bone and C1LMS-R-Con should always be kept in mind if C1LMS is selected for the treatment of upper cervical disorders, with which an increased O-C2 angle is especially associated, and careful postoperative follow-up is mandatory for early detection of OcBE in such cases. From the viewpoint of the surgical procedure, it might be better to insert C1LMS directly on the lateral mass by Goel et al.’s method, and the unnecessary protruded end of C1LMS-R-Con should be as short as possible to prevent the occurrence of OcBE.
